# Boosting Specific Energy and Power of Carbon-Ionic Liquid Supercapacitors by Engineering Carbon Pore Structures

**DOI:** 10.3389/fchem.2020.00006

**Published:** 2020-02-18

**Authors:** Dong Zhang, Hongquan Gao, Guomin Hua, Haitao Zhou, Jianchun Wu, Bowei Zhu, Chao Liu, Jianhong Yang, De Chen

**Affiliations:** ^1^School of Materials Science and Engineering, Jiangsu University, Zhenjiang, China; ^2^Department of Chemical Engineering, Norwegian University of Science and Technology, Trondheim, Norway

**Keywords:** hierarchical porous carbon, slit-pore structure, ion-packing density, ionic liquid, supercapacitors

## Abstract

Carbon-ionic liquid (C-IL) supercapacitors (SCs) promise to provide high capacitance and high operating voltage, and thus high specific energy. It is still highly demanding to enhance the capacitance in order to achieve high power and energy density. We synthesized a high-pore-volume and specific-surface-area activated carbon material with a slit mesoporous structure by two-step processes of carbonization and the activation from polypyrrole. The novel slit-pore-structured carbon materials provide a specific capacity of 310 F g^−1^ at 0.5 A g^−1^ for C-IL SCs, which is among one of the highest recorded specific capacitances. The slit mesoporous activated carbons have a maximum ion volume utilization of 74%, which effectively enhances ion storage, and a better interaction with ions in ionic liquid electrolyte, thus providing superior capacitance. We believe that this work provides a new strategy of engineering pore structure to enhance specific capacitance and rate performance of C-IL SCs.

## Introduction

As the portable energy storage market grows, such as the grid energy storage of electric vehicles and renewable energy, it becomes urgent to develop energy storage devices possessing high specific power and high specific energy (Armand and Tarascon, 2008; Simon and Gogotsi, 2008; Fang et al., 2018; Yun et al., 2019a; Zou et al., 2019). A supercapacitor (SC), a safe and reliable energy storage device with fast charge–discharge capability and a long cycling life, is a competitive energy storage option to meet the increasing power demands (Winter and Brodd, 2004; Aricò et al., 2005; Miller and Simon, 2008; Miller, 2012; Hou et al., 2015; Li et al., 2019; Yun et al., 2019b). However, compared with various batteries (60–200 Wh kg^−1^), the wide application of commercial activated carbon–based SCs has been limited due to their low specific energy (<5 Wh kg^−1^; Aurbach et al., 2000; Burke, 2000; Jang et al., 2011; Zhou et al., 2018). Therefore, without sacrificing the specific power of the SCs itself, increasing its specific energy is the ultimate method to address the problem.

The specific energy density of an SC can be calculated by the equation *E* = *CV*^2^/2 (Miller, 2012). Therefore, the energy density can be increased by developing a high capacitance electrode material or enlarging its voltage window. In particular, it is more remarkable to increase the specific capacitance of SCs. Therefore, the preparation of a carbon-based SC with a high ion-accessible specific surface area (SSA) will improve its capacitance greatly. However, this is not the case when the SSA of the material is very large (>2,000 m^2^ g^−1^). For instance, the maximum SSA of activated carbon reported in the previous literature was limited at about 4,000 m^2^ g^−1^, but the material showed a relatively low specific capacitance (165 F g^−1^) in aqueous electrolyte (To et al., 2015). It can be seen that the specific capacitance is also related to the ion-accessible holes. Chmiola et al. (2006) demonstrated that anions and cations in the organic electrolyte can enter the microporous region [pore size ≈ ion size (d_ion_) of electrolyte] to obtain the highest specific capacitance. Recently, Wei et al. have prepared a specific microporous activated carbon electrode by a chemical activation method, and this kind of activated carbon exhibits an ultra-high specific capacitance of 200–300 F g^−1^ in an organic electrolyte or an ionic liquid (IL) (Wei et al., 2011, 2012). Extremely narrow micropores have been proven to possess optimized high capacitance. However, as with the IL electrolyte with a high voltage window used, the power characteristics of extremely narrow micropores are still limited, especially under the condition of high specific power. With such a limitation mainly due to the large ionic size of IL, the ion transport resistance in the IL electrolyte is high, which causes a rapid decrease in capacitance, thereby affecting the rate performance of the SC (Kondrat et al., 2012; Merlet et al., 2012; Peng et al., 2014; Wen et al., 2016). Chen et al. have found that the pore size region of the favorable capacitors, except for the micropore region, is the mesoporous region (pore size ≈ 2 to 3.5 times ion size of IL; Wang et al., 2016b). Compared with the micropores, the mesopores are also more favorable for rapid ion migration and improving the rate performance of the IL-based SC. Due to the model-confined favorable ion packing, the rich mesopore nanocarbon materials synthesized in this study delivered high capacitance (290 F g^−1^). Chen and coworkers also proposed a model to describe the confined ion packing in the cylindrical pores and pointed to the importance of the three-dimensional (3D) structure of the pores, instead of only a 2D surface, in determining the capacitance of the carbon-IL SCs. The higher mesopore volume and reasonable mesoporous pore size distribution (PSD) not only increase the specific capacitance of the material but also reduce the resistance during electrolyte ion transport, resulting in superior rate capability. However, the capacitance and pore volume relationship needs experimental validation. In addition, based on the analysis of the 3D pore–IL interaction, not only the PSD and pore volume but also the pore morphology could play a privileged role for capacitance. However, the possible effect of the pore morphology on the capacitance has not been addressed so far.

Herein, we focus on the effect of the pore properties such as pore morphology, pore volume, and PSD on the capacitance and rate capability of carbon-IL SCs. A high-pore-volume and specific-surface-area activated carbon material with a slit-pore structure was synthesized by two-step process of carbonization and activation. A series of spherical porous carbon materials was synthesized, namely, carbon nanospheres (CNSs), by using a polypyrrole (PPy) as the carbon source. The PPy after carbonization was activated by KOH at different temperatures. By changing the activation temperature, the regulation of the PSD of the activated carbon material and the increase of the mesopore volume ratio are realized. Superior performance of CNSs-IL SCs is achieved by slit-pore morphology, large pore volume, and proper PSD.

## Experiment

### Synthesis of the CNSs

A solution of 1 M hydrochloric acid (HCl) (37%) solution containing 4.04 g of pyrrole (Aladdin 99%) was mixed at a volume ratio of 1:1 with another solution of 1 M HCl containing 13.74 g of ammonia peroxydisulfate (Aladdin 98%) oxidant. After the mixture was stirred at 500 rad min^−1^ for 2 h, the PPy was filtered and washed with water and ethanol and then dried in a vacuum overnight at 60°C. Then, the synthesized PPy was carbonized at 650°C with a heating rate of 10°C min^−1^ for 2 h in a quartz tube furnace under an argon atmosphere. The carbonized PPy (CPPy) and potassium hydroxide (KOH) pellets (Aladdin 95%) were then ground in ethanol at a mass ratio of 1:4. The mixture was then activated between 800 and 900°C. The heating rate was 5°C min^−1^, and the maximum temperature was maintained for 1 h. Therefore, the PPys activated (referred to as APPy) at 800, 850, and 900°C were named APPy-800, APPy-850, and APPy-900, respectively. The synthesized APPy samples were washed repeatedly with 1 M HCl solution, distilled water, and ethanol until pH was 7. Finally, the samples were dried overnight at 60°C in air.

### Material Characterization

The morphology information and microstructures of the synthesized samples were determined by field-emission scanning electron microscopy (FE-SEM, JSM-7800F) and high-resolution transmission electron microscopy (HR-TEM, Tecnai G2 F30). Brunauer–Emmett–Teller (BET) SSA and PSD of the CNSs were studied by a Micromeritic BEL, BELSORP-MAX instrument. X-ray photoelectron spectroscopy (XPS, Thermo ESCALAB 250XI) was carried out for analysis of the surface chemical composition. X-ray diffraction (XRD) analysis was conducted by a Bruker AXS D8 Discover diffractometer with the Cu K_α_ radiation (λ = 0.1540598 nm). In addition, the carbon materials were investigated by Raman spectroscopy (Renishaw inVia) with a laser wavelength of 532 nm.

### Electrochemical Characterization

The electrode materials were prepared by mixing 80 wt.% APPy, 10 wt.% acetylene black, and 10 wt.% polytetrafluoroethylene (PTFE) in a small amount of ethanol. The obtained electrode materials of the SCs were coated over a nickel foam disk (Alfa Aesar) and pressed at 6 MPa. The mass of the coated active material was approximately 2 mg cm^−2^. Then the electrodes were dried at 120°C for 12 h in a vacuum oven. Finally, the two-electrode cells were assembled using CR2025 coin cells in an argon-filled glove box (<1 ppm O_2_ and <1 ppm H_2_O). The two electrodes in the coin cells were separated by a thin microporous monolayer membrane (Celgard 3501) separator and filled with 15 ml of neat 1-ethyl-3-methylimidazolium tetrafluoroborate (EMIMBF_4_) (Sigma-Aldrich 99%) electrolyte.

The prepared CR2025 coin cells (SCs) were stably placed overnight at room temperature before electrochemical measurements were taken. Impedance spectra (EIS) of the electrodes recorded in a frequency range from 10 to 100 kHz with a voltage amplitude of 10 mV at the open-circuit potential and cyclic voltammetry (CV) of the electrodes were carried out on a Princeton VersaSTAT potentiostat analyzer. Galvanostatic charge–discharge (GCD) tests and cycling stability tests were performed on an MTI S eight-channel battery analyzer. The specific capacitance, specific energy, and specific power of the cells were calculated from the GCD measurements by using Equations S1–S3 (as seen in the Supplementary Material).

## Results and Discussion

**Figure 1:**
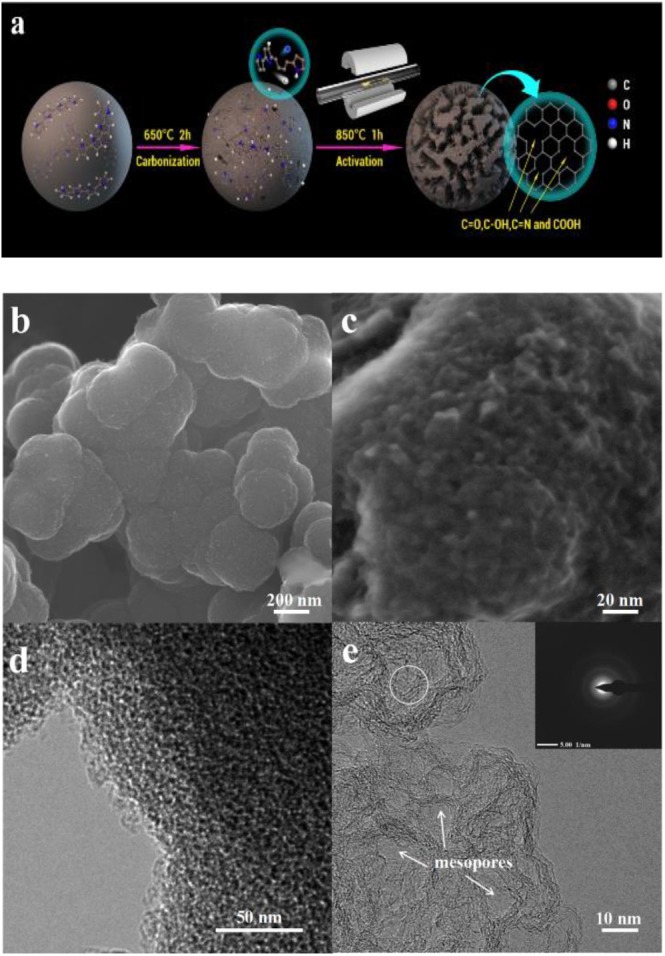
**(a)** Schematic illustration of the synthesis of polypyrrole (Ppy)-derived porous carbon materials. **(b,c)** Scanning electron microscopy (SEM) image of PPy activated at 850°C (APPy-850). **(d,e)** Transmission electron microscopy (TEM) image of APPy-850.

We prepared PPy-derived porous carbon by a carbonization–activation process (Figure 1a). This preparation of porous carbon by a two-step process has a range of physical properties depending on the carbon source and the corresponding activation process. In the present work, the microstructure and morphology information of the synthesized PPy, CPPy, and APPy-850 was characterized using FE-SEM and HR-TEM. The image shows the agglomerates composed of 3D spherical PPys (Figure S1A) with diameters of 100–200 nm. After pyrolysis of the PPy at 650°C for 2 h (Figure S1B), the diameters of the sphere remain unchanged. The surface of the sphere becomes rough after activation at 850°C (Figure 1b), and uniformly distributed mesopores can be observed on the macroporous framework based on high-magnification SEM (Figure 1c) and HR-TEM (Figure 1d) observation. The image (Figure 1e) clearly shows that CPPy is etched with KOH during the activation process, thus obtaining a distribution of micro- and sub-mesopores. It is also shown that the electron diffraction photograph in the inset of Figure 1d confirms the graphitic domain structure (circled area in Figure 1e; Xu et al., 2019). The micro- and sub-mesopores are mainly caused by the delamination of the graphitic domain. After activation, these domains are delaminated, and slit-shaped pores are produced. When the degree of delamination is high, cylindrical or conical pores form. A large number of slit-shaped pores formed in APPy-850 are apparent in Figure S1C (circled area). Simultaneously, such a hierarchical pore structure allows the IL to be better stored and transferred inside the electrode.

**Figure 2:**
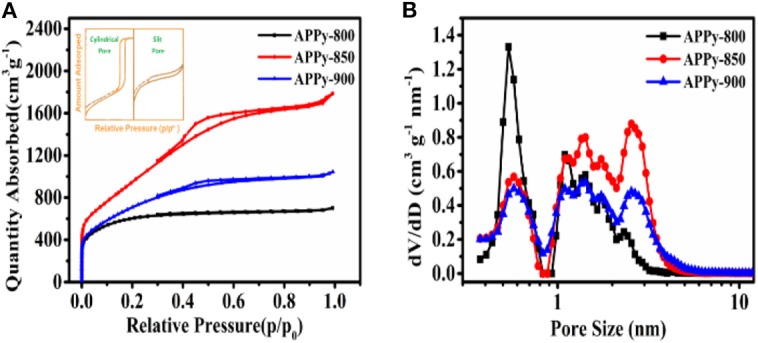
**(A)** Nitrogen adsorption/desorption isotherm at 77 K. **(B)** Pore size distributions calculated using a slit-pore density functional theory (DFT) model.

The N_2_ adsorption/desorption method was used to measure the pore characteristics of the APPys obtained at different activation temperatures. Although the SSA obtained from the BET method is not rigorous (Rouquerol et al., 2007), herein, the BET method is used to evaluate the relative surface area of different samples. The results indicate that the activation temperature has a significant effect on the SSA, the pore structure, and PSD (Figures 2A,B). The SSA of APPy-800, APPy-850, and APPy-900 are 2207.1, 3818.8, and 2620.5 m^2^ g^−1^, respectively.

Regarding the specific pore structure in the APPys, the hysteresis loop shapes have often been identified with specific mesopore structures. The hysteresis loop made by slit pores seems nearly horizontal and parallel over a wide range of p/p_o_. And the hysteresis loop made by cylindrical pores is almost vertical and nearly parallel over an appreciable range of gas uptake (Sing, 1985; Thommes et al., 2000, 2002). Illustrated hysteresis loops corresponding to cylindrical pores and slit pores are presented as an insert in Figure 2A. As presented in Figure 2A, the hysteresis loop between the N_2_ adsorption and desorption isotherms indicates that the porous carbon materials mainly contain slit mesopores, rather than cylindrical pores. Furthermore, the mesopore content in the porous carbon materials can be evaluated by the size of the hysteresis loop (Sing, 1982). In general, a larger hysteresis loop indicates a high proportion of mesopores, and a small hysteresis loop implies a low proportion of mesopores. By comparison of the hysteresis loops of three porous carbon materials, APPy-800, APPy-850, APPy-900, it can observed that APPy-850 has the highest content of mesopores.

According to the non-local density functional theory (NL-DFT) based on the slit-pore model, the pore structure parameters of APPys are listed in Table S1. It can be observed that the pore volumes of APPy-800, APPy-850, and APPy-900 are 0.987, 2.098, and 1.487 cm^3^ g^−1^, respectively. The PSD of APPys are presented in Figure 2B; it can be seen that pore volumes of APPy-850 and APPy-900 increase in the range of mesopore size and decrease in the range of micropore size in comparison to APPy-800. The narrow small mesopore size ranges from 2 to 5 nm. The ratios of mesopore volume to total pore volume in APPy-850 and APPy-900 samples are 72 and 68%, respectively. In addition, it can be observed that the activation process at high temperature (900°C) decreases SSA and pore volume, which is detrimental to achieving high-performing porous carbon materials. Such degradation may be due to excessive temperature, which makes the ablation of C atoms in the KOH activation process more serious, leading to collapse of the pores (Xu et al., 2014).

**Figure 3:**
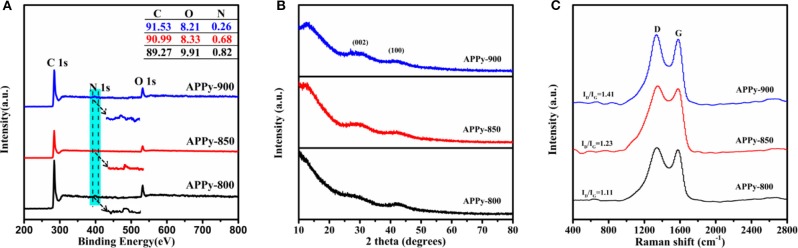
**(A)** X-ray photoelectron spectroscopy (XPS) spectra, and inset is a table of detailed elements in materials. **(B)** X-ray diffraction (XRD) patterns of the APPy porous carbons. **(C)** Raman spectra of the APPy porous carbons.

**Figure 4:**
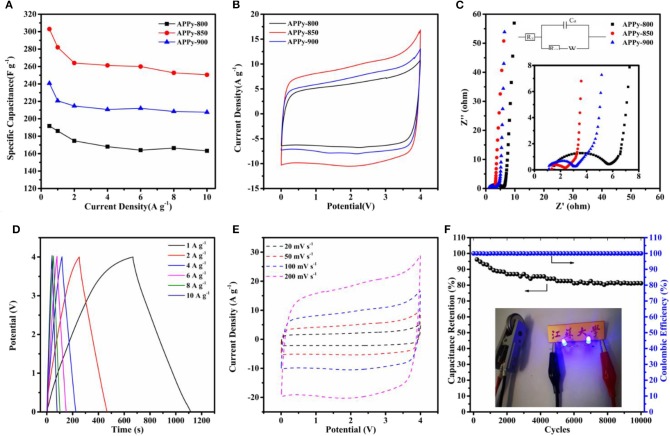
**(A)** Rate capability: current density vs. specific capacitance. **(B)** Cyclic voltammetry (CV) curve of three samples at 100 mv s^−1^. **(C)** Nyquist plots of three samples; the inset is a magnification of the high-frequency range data and fitting equivalent circuit. **(D)** Galvanostatic charge–discharge (GCD) curve of APPy-850-SC at current densities of 1, 2, 4, 6, 8, and 10 A g^−1^. **(E)** CV curve of APPy-850-SC at a scan rate of 20–200 mV s^−1^. **(F)** Cycling stability and coulombic efficiency of APPy-850-SC at 6 A g ^−1^ for 10,000 cycles. The inset shows photographs of two blue LEDs.

The chemical compositions of APPy C1s, O1s, and N1s signals are analyzed by the XPS spectra, and the surface composition of carbons is shown in the XPS survey spectra (Figure 3A and Table S2). From the XPS survey, the samples of APPy-850 are mainly made up of 90.99 at.% carbon with 8.33 at.% oxygen and 0.68 at.% nitrogen, with no other heteroatoms being observable. The high-resolution C1s spectra of APPy (Figure S2) can be deconvoluted by five peaks, representing C-I (284.6 eV), a dominated component for graphitized carbon; C-II (286.0–286.3 eV), carbon in phenolic, alcohol, ether, or C=N groups; C-III (287.3–287.6 eV), carbon in carbonyl or quinine groups; C-IV (288.8–289.1 eV), carbon in carboxyl or ester groups; and C-V (290.5–291.2 eV), carbon in carbonate groups and/or adsorbed CO and CO_2_ (Zhou et al., 2007, 2019). The high-resolution O1s core level spectra can be mainly deconvoluted by four peaks, representing O-I (531.4 eV), C=O quinone-type groups, O-II (532.5 eV), C-OH phenol groups and/or C-O-C ether groups, O-III (533.4 eV), non-carbonyl (ether-type) oxygen atoms in esters, and O-IV (534–535 eV), anhydrides groups and COOH carboxylic groups (Figure S2; Li et al., 2014). It is widely accepted that the surface redox reaction between oxygen functionalities on carbons and electrolyte ions can contribute to the pseudocapacitance through the quinone/hydroquinone redox pair (about 60% of total oxygen; Liu et al., 2016). It is confirmed that in non-aqueous electrolytes, the reversible oxidation/reduction of hydroquinone/quinone and carbonyl (C=O) groups may cause pseudocapacitance (Gupta and Linschitz, 1997). So a small peak of pseudocapacitance at ~2 V can be observed on the discharge section of the CV curve of APPy-850-SC (Figures 4B,E). The nitrogen content in the three APPy samples is relatively low (<0.82 at.%). These traces of N atoms may be doped into carbon materials to enhance the wettability of the interface between the electrode and electrolyte (Li et al., 2007).

The structural features of the porous carbon were further evaluated by using XRD. The XRD pattern (Figure 3B) shows two discernible broad peaks at 25–30°, which corresponds to the (002) plane, and 42–44°, corresponding to the (100) plane of graphite, respectively (Jiang et al., 2013). The broad (002) XRD peaks confirm the low degree of order in these carbons, which is understandable since they are both carbonized and activated at low temperatures. A large increase in the low-angle scatter from the porous carbon can be also noted, which is consistent with the presence of a high density of pores. These results are consistent with the observations from HR-TEM (Figure 1e), which indicate that APPy porous carbon consists predominantly of curved monoatomic layers of carbon, forming a large number of slit-shaped pores between the interlaced flakes (arrows in Figure S1C). The two characteristic G- and D-bands of the Raman spectrum of APPy materials are shown at ~1,595 and ~1,350 cm^−1^, respectively (Figure 3C; Tsai et al., 2013). The former corresponds to the graphitic order, while the latter corresponds to the degree of disorder/defectiveness in the structure. As the activation temperature increases, the I_D_/I_G_ ratio is gradually increased from ~1.11 in APPy-800, ~1.23 in APPy-850 to ~1.41 in APPy-900, indicating an increase of the degree of disorder/defectiveness in the structures. According to XRD diffraction and the Raman spectrum, it can be confirmed that the obtained porous carbon materials consist of stacking graphene-like layers. Intriguingly, as the activation temperature increases, the I_D_/I_G_ ratio is gradually increased from ~1.11 in APPy-800, ~1.23 in APPy-850 to ~1.41 in APPy-900, indicating an increase of the disorder degree in the structures. This is a little controversy to the general observation. Such a discrepancy can be attributed to the activation temperature. When the activation temperature is below 900°C, the increase of activation temperature will lead to poor graphitization degree (Yan et al., 2020).

The GCD measurement results are in the range of 0.5–10 A g^−1^, which confirmed the superior capacitance performance of APPy, as shown in Figure 4A. The APPy-850 shows a high gravimetric capacitance of 310 F g^−1^ (0–4 V) at 0.5 A g^−1^ and high capacitance retention of 83% up to 10 A g^−1^. The excellent performance of this sample is attributed to the large specific surface area and high pore volume, contributed mainly by the rich mesoporous structure. This structure facilitates the rapid transport of ions and thus contributes to high rate performance (Guo et al., 2013). The CV curves of the three APPy samples at the same scan rates (100 mV s^−1^) are shown in the fully operated voltage ranging from 0 to 4 V (Figure 4B). Among them, the APPy-850 shows a larger CV area, indicating that it possesses a higher specific capacitance. The Nyquist plots to investigate the electrochemical behavior of the three APPy samples are shown in Figure 4C, and the inset is an EIS plot at high-frequency region and equivalent circuit. The impedance spectrum mainly consists of a semicircle in the high-frequency region and an approximate straight line in the low-frequency region, where the behavior is mainly capacitive. The 45° segment in the Nyquist plot may be due to the inhomogeneity of the internal pores, indicating that the transport of electrolyte ions during charge and discharge is controlled by the diffusion process (Manohar et al., 2008; He and Mansfeld, 2009). The semicircle reflects the resistance during charge transfer and is related to the porous structure of the electrode (Gamby et al., 2001). From the inset of Figure 4C, the amplitude of the semicircle of the APPy-850 is much smaller than that of the others, indicating that the APPy-850 shows the capacitive behavior at lower resistance values than others. That means the impedance of the electrode with the APPy-850 is less dependent on frequency, and thus, the pore ion accessibility of the APPy-850 is higher than that of the others, indicating that the activation temperature of 850°C provides the best porosity structure for IL ion diffusion.

For the optimized APPy-850, the charge/discharge curve (Figure 4D and Figure S3) still shows a quasi-triangle, indicating excellent electrochemical reversibility and charge/discharge efficiency (Stoller et al., 2008). As shown in Figure 4E and Figure S3, the CV exhibits an approximately rectangular shape, confirming that the capacitance obtained is mainly due to the electric double layer capacitance contribution. Even at a high scanning rate of 200 mV s^−1^, its rectangular shape is maintained, indicating excellent current response capability. This good current performance is due to the structure in which the mesopores and the micropores are cross-linked with each other can store a large amount of electrolyte ions and effectively shorten the ion transport time (Chen et al., 2012; Wang et al., 2016a). Apart from high capacitive performance, long-term cyclic performance is another important factor for a superior energy storage device. The cells were investigated by GCD at a current density of 6 A g^−1^ within the voltage range of 1.8–3.6 V (Figure 4F). After 10,000 cycles, it still maintains about 81% of the initial capacitance, indicating that APPy-850 porous carbon is a well-stabilized SC electrode material (Zhang et al., 2015). Since there is still sufficient free space between the mesoporous structures of these porous carbons, the volume expansion due to prolonged ion insertion/extraction is effectively buffered. Two devices also were connected to light emitting diodes (LEDs, 3.6 V), and the two blue LEDs were successfully lighted up (the inset of Figure 4F) for over 10 min after charging for 15 s.

**Figure 5:**
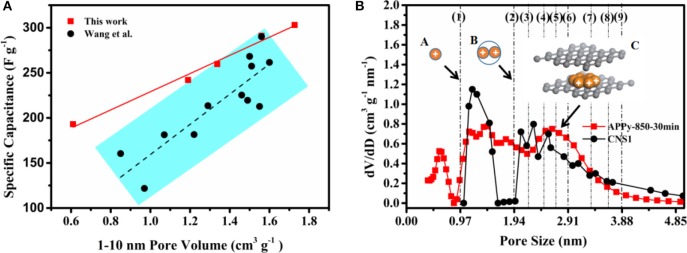
**(A)** Relationship between pore volume and specific capacitance, **(B)** Pore size distribution curve calculated using different models and charge ion-packing configurations. Note: CNS1 stands for carbon nanosponge (CNS), which carbonized at 650°C, followed by activation at 900°C for 60 min. The data of CNS1 are cited from Wang et al. (2016b).

According to the specific capacitance data measured by GCD in Table S1, Figure 5A reveals that the increase in the effective slit-pore volume of APPys between the slit-pore diameters of 1 and 10 nm can lead to the increase in the specific capacity. The specific capacitance increases linearly from 193 to 310 F g^−1^, as the slit-pore volume increases from 0.61 to 1.727 cm^3^ g^−1^. This result shows that the specific capacity is novel linearly proportional to the slit-pore volume. Regarding the relationship between pore volume and specific capacity, a cylindrical pore model has recently been used to interpret such a relationship in terms of Equation 1 and Equation S4 (Wang et al., 2016a)

(1) $$\begin{array}{r} N_{total}=\sum_{D_{1}}^{D_{i}}\left[V\left(D_{i}\right)\cdot f\left(\phi \left(D_{i}/d_{ion}\right),d_{ion}\right)\right]\left(i=1,2,3\ldots n\right) \end{array}$$

where *N*_*total*_ is the number of adsorbed ions, *V* is the specific pore volume, *f* is the ion-packing function, Φ is a function of ion volume utilization fraction (Φ = the volume of the adsorbed ions/the total volume of the pore), *d*_*ion*_ is effective ion size (0.97 nm), and D is the pore size. Due to the ion volume utilization fraction, Φ is a function of D/d_ion_ in the cylindrical pore model, so the increase in specific capacity is not a perfect linear dependence on the increase in cylindrical pore volume. However, the novel slit-pore structure carbon materials in this work give rise to a linear dependence of specific capacity on slit-pore volume; it implies that the ion volume utilization fraction Φ is a constant rather than a function of D/d_ion_. Such a difference in the ion volume utilization fraction Φ indicates that the ion-packing configuration in the novel slit-pore structure is different from that in cylindrical pore structure.

In the following, the difference of ion-packing configuration between a cylindrical pore structure and a slit-pore structure will be investigated. Figure 5B shows PSD curves of the two materials. The PSD curves of CNS1 are calculated using a cylindrical pore DFT model with the pore volume of 1.5 cm^3^ g^−1^ between the pore diameters of 1 and 10 nm. The PSD curves of APPy-850-30 min are calculated using a slit-pore DFT model with the pore volume of 1.56 cm^3^ g^−1^ between the pore diameters of 1 and 10 nm. The PSD in the region of 1–5 nm mainly contributing to the pore volume is considered in this work. When the pore size is (D/d_ion_ < 1), no ions can access the surface inside the pore. When the pore size can exactly adapt the integer number of ions, the densest-surface ion-packing configurations lead to the highest volume utilization. When the pore size within the range accommodates the two nearest integer number of ions, the volume utilization decreases with the increase of D/d_ion_, as demonstrated by the cylindrical pore model (Hodak and Girifalco, 2003; Mughal et al., 2011, 2012). An example is illustrated as schemes A to B in Figure 5B. In the case of D/d_ion_ equals 1 and 2, the ion volume utilization fractions are 21.2 and 15%, respectively (calculated by ϕ of Equation 1). It is also interesting to note that the volume utilization fractions of other integer number of ions (3, 4, 5, 6, 7, 8, and 9) are not higher than the case where the ion-packing number is 1 in the range of 1–5 nm. The densest-surface ion-packing configuration of each layer in the surface of the slit pore has only a hexagonal close-packed (HCP) mode in scheme C of Figure 5B, and the layer of IL ions having an HCP mode will stack up layer by layer as the slit-pore size increases. The HCP structure of ions in the slit pore has the highest volume utilization (74%) (Szczurek et al., 2014). It is evident that a slit-pore structure is superior to the conventional cylindrical pore structure, and it can accommodate more electrolyte ions at an identical pore volume. Therefore, in this work, the slit-pore structures of the APPy materials increase the specific capacitance of the material in the same volume.

**Figure 6:**
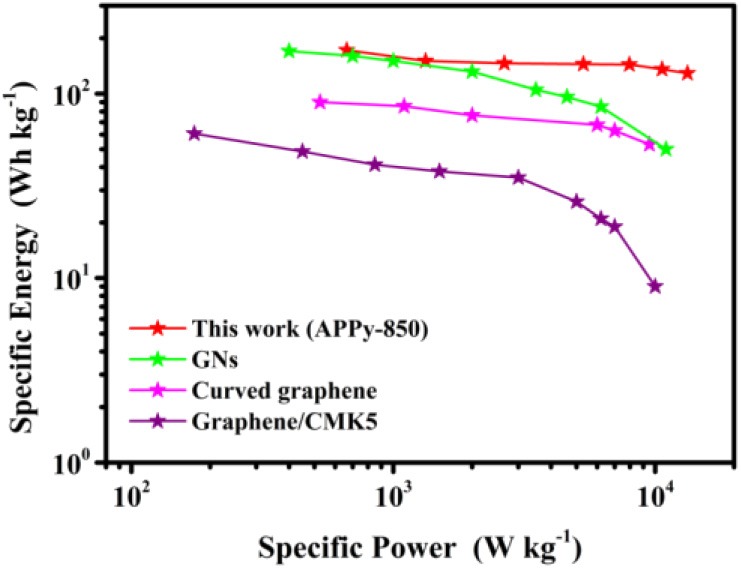
Ragone plots of APPy-850–based electrode compared with those of typical cathode materials based on 1-ethyl-3-methylimidazolium tetrafluoroborate (EMIMBF_4_) electrolytes (Liu et al., 2010; Lei et al., 2013; Gao et al., 2017).

To further confirm the superior performance of our SC with an APPy-850–based electrode, the Ragone plot for APPy-850 material is compared with other electrode materials in Figure 6. Based on the active mass in the electrode, the APPy-850-SC possessed an excellent specific energy of 171.5 Wh kg^−1^ at the specific power of 664 W kg^−1^. Even at a much higher specific power of 15 kW kg^−1^, the APPy-850-SC still maintains a high specific energy of 129.1 Wh kg^−1^. In Table S3, the performance of the current system is compared with other SCs based on the same IL electrolyte. The APPy-850-SC exhibits significantly higher specific capacity and remains quite competitive with other performances.

## Conclusions

Here we have shown a privilege role of the ion-packing density in determining the performance of carbon-IL SCs. We have shown that the specific capacity of the porous material is linearly proportional to the effective pore volume and is also closely related to the pore structure. We report that the 2D slit-pore structure with the HCP configuration of ions has the highest ion-packing density. The slit-pore structure has superior ion-packing density compared to cylinder-like pores. As a result, optimized synthetic APPy-850 hierarchical porous carbon with a high pore volume and a rich slit mesoporous structure achieved a specific capacity of 310 F g^−1^ at 0.5 A g^−1^ for IL SCs, which is one of the highest recorded specific capacitances. At the same time, high specific energy and excellent cycling performance at high specific power are also shown. This work provides a new idea to increase the specific capacity and rate performance of carbon materials by increasing the volume fraction of the slit-type mesopores, which makes it potentially possible to develop SCs with higher specific energy and specific power.

## Data Availability Statement

All datasets generated for this study are included in the article/Supplementary Material.

## Author Contributions

All authors listed have made a substantial, direct, and intellectual contribution to the work, and approved it for publication.

### Conflict of Interest

The authors declare that the research was conducted in the absence of any commercial or financial relationships that could be construed as a potential conflict of interest.
