# Supplementary material for: Boosting Specific Energy and Power of Carbon-Ionic Liquid Supercapacitors by Engineering Carbon Pore Structures
Source: Front Chem. 2020 Feb 18;8:6. doi: 10.3389/fchem.2020.00006 (PMC7040027; doi:10.3389/fchem.2020.00006)
Supplement: Supplementary file 1 [file Data_Sheet_1.pdf]

Supplementary Material

The specific capacitance (or gravimetric capacitance) of a single electrode was calculated from the GCD discharge curve according to:

$$C_{sp,electrode} = \frac{4I_{cons} \cdot \Delta t}{m \cdot V_{\max}} \qquad S1$$

where I_{cons} is the constant discharge current, m is the total mass for both active materials on the electrode, Δt is the discharge time and V_{max} is the cell voltage change during the discharge process (excluding the voltage drop at the beginning of the discharge).

The specific energy and the average specific power of the cell were estimated:

$$E_{sp,cell} = \frac{C_{sp,electrode} \cdot V_{\max}^{2}}{8} \qquad S2$$

$$P_{sp,cell,ave} = \frac{E_{sp,cell}}{\Delta t} \qquad S3$$

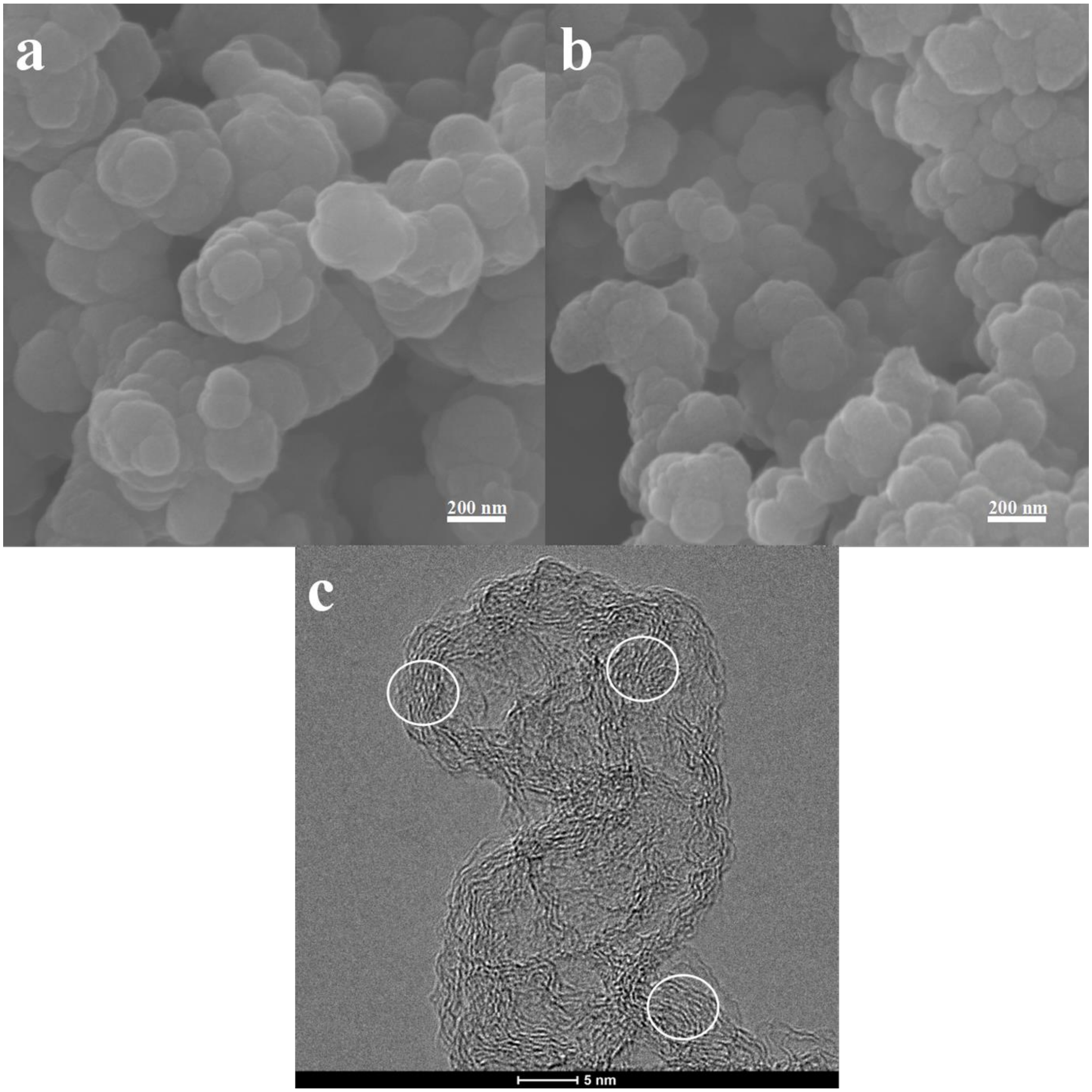

Figure. S1 SEM image of PPy (a), CPPy (b), TEM image of APPy-850 (c).

Table S1. Pore characteristics of all simples.

Sample	BET SSA ($m^2 g^{-1}$)	T-plot SSA ($m^2 g^{-1}$)	surface area ($m^2 g^{-1}$)		pore volume ($cm^3 g^{-1}$)		Csp,electrode at 0.5 A g^{-1} (F g^{-1})
			NLDFT	1-10 nm	NLDFT	1-10 nm	
APPy-800-1h	2207	2238	1243	693	0.987	0.610	193
APPy-850-30min[a]	3585	2830	1733	1376	1.945	1.560	291
APPy-850-1h	3818	2951	1832	1471	2.098	1.727	310
APPy-850-2h[b]	2579	2202	1349	1014	1.669	1.337	259.7
APPy-900-1h	2620	2252	1375	1000	1.487	1.190	242

Note:[a b] the APPy-850-30min and APPy-850-2h were prepared at 850 °C for 30min and 2h, respectively.

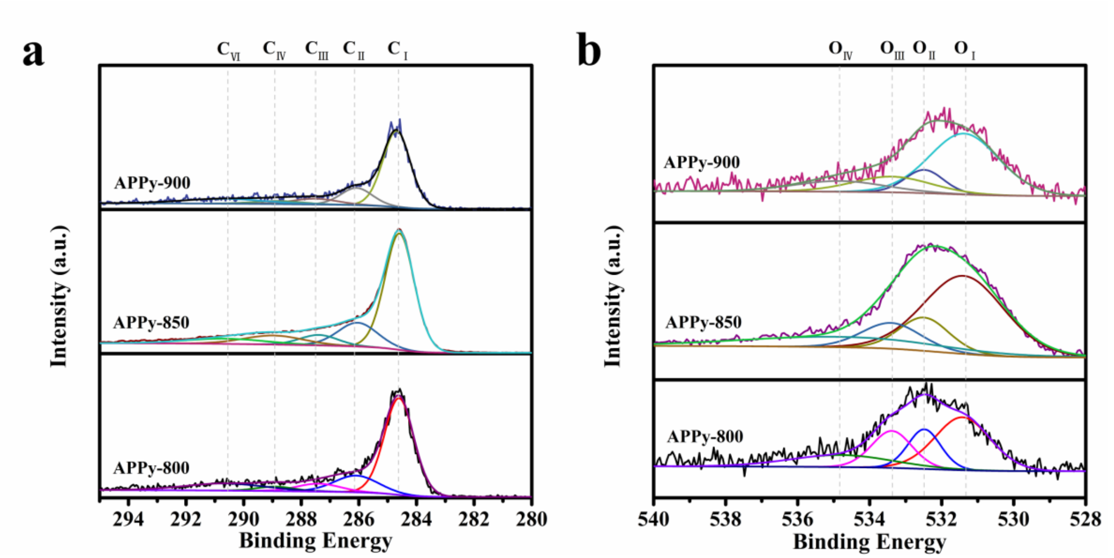

Figure. S2 High-resolution XPS C1s spectra (a) and O1s spectra (b).

Table S2. Summarization of different functional groups on the surface of CNSs.

Atom type	Peak label	Peak position/ eV	Group	Percentage (%)		
				CPPy-800	CPPy-850	CPPy-900
C 1s	C_I	284.6	Graphitized carbon	60.95	63.37	60.45
	C_{II}	286.1	C–OH	15.67	13.57	15.73
	C_{III}	287.4	C=O	7.37	8.05	7.70
	C_{IV}	288.9	O=C-O	3.61	7.37	4.47
	C_V	290.6	Carbonate groups	15.40	7.64	11.65
O 1s	O_I	531.4	C=O	42.14	54.47	57.43
	O_{II}	532.5	C=O and C–OH	18.65	14.42	12.41
	O_{III}	533.4	C-O-C and C–OH	21.45	13.20	10.23
	O_{IV}	534.8	C=O in carboxyl	17.81	17.91	19,93

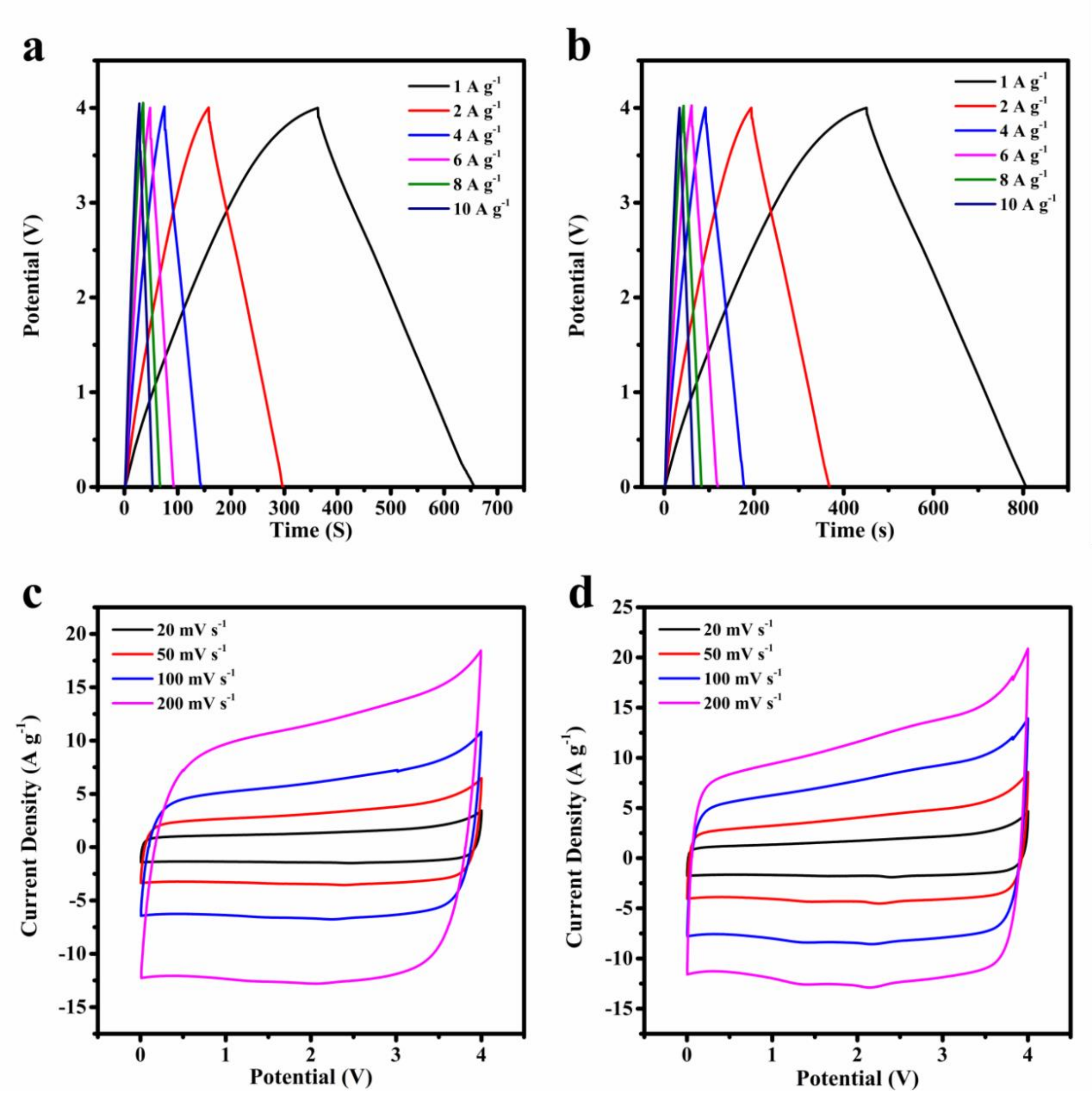

Figure. S3 GCD curve of (a) APPy-800-SC, (b) APPy-900-SC at current densities of 1, 2, 4, 6 ,8 and 10 A g^{-1}, CV curve of (c) APPy-800-SC, (d) APPy-900-SC at a scan rate of 20–200 mV s^{-1}

The gravimetric capacitance of a single electrode can be expressed as:(Wang et al., 2016b)

$$C'_{sp,electrode} = \frac{Q}{U} = \frac{2e \cdot N_{total}}{U} \quad \text{S4}$$

where Q is the total charge on the electrode, U is the potential absolute value of the single electrode, and e is one electron charge equals to 1.6×10^{-19} C.

Table S3. Comparison of specific capacitance, specific energy, specific power and stability of various capacitor systems with the current work.

Material	Electrolyte	Specific Capacitance ($F\ g^{-1}$)	Specific energy ($Wh\ kg^{-1}$)	Specific power ($W\ kg^{-1}$)	Retention (%)	Cycles	Refs.
APPy-850 (This work)	$EMIMBF_4$ (0-4 V)	310 ($0.5A\ g^{-1}$)	171.5	664	81 ($6A\ g^{-1}$)	10000	
Curved graphene	$EMIMBF_4$(0–4 V)	154 ($1\ A\ g^{-1}$)	90	525	Not studied	Not studied	(Liu et al., 2010)
Graphene/CMK-5	$EMIMBF_4$(0-3.5 V)	145 ($0.2\ A\ g^{-1}$)	60.7	174		2000	(Lei et al., 2013)
Glucose derived carbon	$EMIMBF_4$(0-3.2 V)	158 ($1\ mV\ s^{-1}$)	45	130	Not studied	Not studied	(Tooming et al., 2015)
GNs	$EMIMBF_4$(0–4 V)	306 ($0.5A\ g^{-1}$)	170	400	73.5 ($5A\ g^{-1}$)	10000	(Gao et al., 2017)
PANI nanofiber	$EMIMBF_4$(0–4 V)	291 ($0.1A\ g^{-1}$)	162	100	70 ($10A\ g^{-1}$)	10000	(Wang et al., 2016a)

References

Gao, B., Zhou, H., and Yang, J. (2017). One-step preparation of nitrogen-doped graphene nanosheets for high-performance supercapacitors. *Applied Surface Science* 409**,** 350-357. doi: 10.1016/j.apsusc.2017.03.015.

Lei, Z., Liu, Z., Wang, H., Sun, X., Lu, L., and Zhao, X.S. (2013). A high-energy-density supercapacitor with graphene–CMK-5 as the electrode and ionic liquid as the electrolyte. *Journal of Materials Chemistry A* 1(6)**,** 2313-2321. doi: 10.1039/c2ta01040b.

Liu, C., Yu, Z., Neff, D., Zhamu, A., and Jang, B.Z. (2010). Graphene-based supercapacitor with an ultrahigh energy density. *Nano letters* 10(12)**,** 4863-4868. doi: 10.1021/nl102661q.

Tooming, T., Thomberg, T., Kurig, H., Jänes, A., and Lust, E. (2015). High power density supercapacitors based on the carbon dioxide activated d-glucose derived carbon electrodes and 1-ethyl-3-methylimidazolium tetrafluoroborate ionic liquid. *Journal of Power Sources* 280**,** 667-677. doi: https://doi.org/10.1016/j.jpowsour.2015.01.157.

Wang, X., Zhou, H., Lou, F., Li, Y., Buan, M.E., Duan, X., et al. (2016a). Boosted Supercapacitive Energy with High Rate Capability of a Carbon Framework with Hierarchical Pore Structure in an Ionic Liquid. *ChemSusChem* 9(21)**,** 3093-3101. doi: 10.1002/cssc.201600779.

Wang, X.H., Zhou, H.T., Sheridan, E., Walmsley, J.C., Ren, D.D., and Chen, D. (2016b). Geometrically confined favourable ion packing for high gravimetric capacitance in carbon-ionic liquid supercapacitors. *Energy & Environmental Science* 9(1)**,** 232-239. doi: 10.1039/c5ee02702k.
